# MEETINGS CALENDAR - 2017

**Published:** 2017

## November

### ► 2 to 4 - 3rd Global Summit on Heart Diseases

Bangkok, Thailand

Informations: Programme Coordinator

Phone: 7025085200

E-mail: heartdisease@cardiologyconference.org

Site: heartdiseases.conferenceseries.com/asiapacific/

### ► 2 and 3 - British and Irish Society for Minimally Invasive Cardiac Surgery Annual Meeting

London, United Kingdom

Informations: L.R. Associates - Ms. L. Richardson

Phone: 077 111 32946

Fax: 01296 733 823

E-mail: lorrainerichardson1@btinternet.com

Site: www.bismics.org.uk

### ► 3 - The Treatment of CAD: Past, Present and Future: Celebrating 50 years of CABG

Cleveland, United States

Informations: Jamie Belkin

Phone: 216 932 3448

E-mail: jamie@jamiebelkin.com

Site: http://www.clevelandclinicmeded.com/live/courses/ich/

### ► 7 to 10 - 28th Annual Cardiovascular Interventions 2017

La Jolla, United States

Informations: Promedica International CME

Phone: 760-720-2263

Fax: 760-720-6263

Site: promedicacme.com/

### ► 8 to 10 - ESTS Course on Medical Writing

Hamburg, Germany

Informations: Sue Hesford

Phone: + 44 1392 430671

E-mail: sue@ests.org.uk

Site: www.ests.org

### ► 8 to 11 - STSA 64th Annual Meeting

San Antonio, United States

Informations: Southern Thoracic Surgical Association

Phone: 312.202.5892

Fax: 773.289.0871

E-mail: stsa@stsa.org

Site: stsa.org/64thannual/

### ► 11 to 12 - Eastern India Cardiac Conclave

Kolkata, India

Informations: Sandip Sardar

E-mail: secretary.eicc@gmail.com

Site: www.cardiacconclave.com

### ► 13 to 14 - Surgery of the Thoracic Aorta - 9th Postgraduate Course

Bologna, Italy

Informations: Monica Falzoni

Phone: +39 051230385

Fax: +39 051221894

E-mail: m.falzoni@noemacongressi.it

Site: www.noemacongressi.it

### ► 13 to 14 - Annual Cardiology Congress

Singapore, Singapore

Informations: Akansha

Phone: 1-302-231-6756

E-mail: cardiology@meetingseries.org

Site: www.meetingsint.com/healthcare-conferences/cardiology/registration

### ► 14 to 17 - Congenital Heart Disease

Windsor, United Kingdom

Informations: EACTS

E-mail: info@eacts.co.uk

Site www.eacts.org/educational-events/programme/2017-congenital-heart-disease/

### ► 15 to 18 - 50th Chilean Society of Respiratory Diseases Conference

Puerto Varas, Chile

Informations: SER Chile

E-mail: ser@serchile.cl

Site: www.serchile.cl

### ► 16 to 19 - 27th Annual Congress of the Association of Thoracic and Cardiovascular Surgeons of Asia (ATCSA)

Melbourne, Australia

Informations: Simone Watt

Phone: +61 3 9249 1158

E-mail: atcsa2017@surgeons.org

Site: www.atcsa2017.com

### ► 16 and 17 - ESTS Course on Terrorism and Disaster Management

Strasbourg, France

Informations: Sue Hesford

Phone: 01392 430671

E-mail: sue@ests.org.uk

Site: www.ests.org

### ► 16 and 17 - Controversies & Advances in the Treatment of Cardiovascular Disease: The Seventeenth in the Series

Beverly Hills, United States

Informations: Promedica International CME

Phone: 760-720-2263

Fax: 760-720-6263

Site: www.controversiesincardiology.com/

### ► 20 to 23 - ESTS Course on TB and other Lung Diseases of Surgical Interest

Cepina, Italy

Informations: Sue Hesford

Phone: + 44 1392 430671

E-mail: sue@ests.org.uk

Site: www.ests.org

### ► 23 to 25 - Thoracic Surgery: Part III

Windsor, United Kingdom

Informations: EACTS

E-mail: info@eacts.co.uk

Site: www.eacts.org/educational-events/programme/

## December

### ► 29 to 2 - 12th European Mechanical Circulatory Support Summit (EUMS)

Bad Oeynhausen, Germany

Informations: EACTS

E-mail: info@eacts.co.uk

Site: www.eacts.org/educational-events/programme/

### ► 1 and 2 - Mastering the Mitral Valve

New York, United States

Informations: Jamie Belkin

Phone: 216 932 3448

E-mail: jamie@jamiebelkin.com

Site: www.ccfcme.org/mitralmasters

### ► 3 to 5 - ICI Meeting 2017

Tel Aviv, Israel

Informations: ICI 2017 Secretariat

Phone: +972-3-5767737/9

E-mail: secretariat@icimeeting.com

Site: 2017.icimeeting.com/

### ► 8 and 9 - AATS International Cardiovascular Symposium

São Paulo, Brazil

Informations: AATS

Phone: 978-252-2200

Fax: 978-522-8469

E-mail: meetings@aats.org

Site: www.aats.org/ics

### ► 11 and 12 - 22nd World Cardiology Conference

Rome, Italy

Informations: Ellena Stewart

Phone: 1-702-508-5200

Ext: 8033

Site: worldcardiology.conferenceseries.com/

